# A Manual for the Practice of Surgery

**Published:** 1886-04

**Authors:** 


A MANUAL FOR THE PRACTICE OF SURGERY. *By* THOMAS BRYANT, F.R.C.S. 1885. *Fourth Edition, etc., etc.* Pp, 1026. *Philadelphia:* HENRY C. LEA'S SON & CO. *Chicago:* JANSEN, MCCLURG & CO.

The fourth edition of this work is fully abreast of the times. The author handles his subjects with that degree of judgment and skill which is attained by years of patient toil and varied experience. The present edition is a thorough revision of those which preceded it, with much new matter added, notably in the chapters devoted to the pathological conditions of the eye and ear, and their treatment. He has also amplified his remarks upon hernia, the diseases of the genito-urinary organs and oral surgery. His definition of the term surgery is concise and comprehensive, viz.: "Surgery is of a two-fold nature; it is a science and also an art,—a department that requires to be known and another to be practiced." His remarks concerning the method of investigating a case are of value to the young surgeon. His diction is so graceful and logical, and his explanations are so lucid as to place the work among the highest order of text-books for the medical student.

Almost every topic in surgery is presented in such a form as to enable the busy practitioner to review any subject in every day practice in a short time. No time is lost with useless theories or superfluous verbiage. In short, the work is eminently clear, logical and practical.

The publication consists of one imperial octavo volume, containing 1,026 pages, and forms an admirable companion-book to Gray's Anatomy, which corresponds with it in size.

